# Multi-metric locality sensitive hashing enhances alignment accuracy of bisulfite sequencing reads: BisHash

**DOI:** 10.1093/bioadv/vbaf144

**Published:** 2025-07-23

**Authors:** Hassan Nikaein, Ali Sharifi-Zarchi

**Affiliations:** Department of Computer Engineering, Sharif University of Technology, Tehran, 1458889694, Iran; Department of Computer Engineering, Sharif University of Technology, Tehran, 1458889694, Iran

## Abstract

**Motivation:**

Locality-Sensitive Hashing (LSH) is a widely used algorithm for estimating similarity between large datasets in bioinformatics, with applications in genome assembly, sequence alignment, and metagenomics. However, traditional single-metric LSH approaches often lead to inefficiencies, particularly when handling biological data where regions may have diverse evolutionary histories or structural properties. This limitation can reduce accuracy in sequence alignment, variant calling, and functional analysis.

**Results:**

We propose Multi-Metric Locality-Sensitive Hashing (M2LSH), an extension of LSH that integrates multiple similarity metrics for more accurate analysis of complex biological data. By capturing diverse sequence and structural features, M2LSH improves performance in heterogeneous and evolutionarily diverse regions. Building on this, we introduce Multi-Metric MinHash (M3Hash), enhancing sequence alignment and similarity detection. As a proof of concept, we present BisHash, which applies M2LSH to bisulfite sequencing, a key method in DNA methylation analysis. Although not fully optimized, BisHash demonstrates superior accuracy, particularly in challenging scenarios like cancer studies where traditional approaches often fail. Our results highlight the potential of M2LSH and M3Hash to advance bioinformatics research.

**Availability and implementation:**

The source code for BisHash and the test procedures for benchmarking aligners using simulated data are publicly accessible at https://github.com/hnikaein/bisHash.

## 1 Introduction

A hash function is a mathematical function that maps inputs of various sizes to a finite output set in a non-uniform manner Knuth (1998). These functions primarily reduce the space needed to store information for searching within the input set. Although hash functions are primarily employed in search algorithms, their sensitivity to even minimal changes in input presents challenges when applying them to similarity measurement and various bioinformatics problems. In applications such as detecting similarity between two reads, especially in the presence of errors, this sensitivity is not only unhelpful but may also lead to reduced accuracy.

A notable category of hash functions that has seen applications in both bioinformatics research and other fields, such as search engine technologies Broder (2000) and image similarity detection Chum *et al.* (2008), is Locality-Sensitive Hashing (LSH) Indyk and Motwani (1998); Gionis *et al.* (1999). These functions are designed to have a low probability of output alteration when there are minor changes in the input.

A locality-sensitive hash function is characterized by its property that small changes to the input result in a low probability of altering the output. In more formal terms, if two inputs have differences below a certain threshold, the output is likely to remain unchanged with a probability above a predetermined threshold. Defining the “difference” between two inputs requires a “metric” to measure it, forming the foundation of a locality-sensitive hash function. For instance, if we consider the Hamming distance between points in a space as the difference between two points, projecting the points onto one of the axes can function as a locality-sensitive hash.

### 1.1 MinHash as a locality-sensitive hashing

MinHash Broder (2000) is a locality-sensitive hash function that utilizes the Jaccard distance as its metric, applied in set space. Its primary purpose is to approximate the Jaccard similarity JAC coefficient, denoted as J(A,B), between two sets *A* and *B*. The Jaccard similarity coefficient is defined as $J(A,B)=\frac{\left|A\cap B\right|}{\left|A\cup B\right|}$ while the Jaccard distance is given by JDIS(A,B)=1−J(A,B).

For two distinct sets, the Jaccard similarity ranges from 0 (disjoint sets) to 1 (identical sets), with intermediate values representing varying degrees of similarity.

To define MinHash, we consider η hash functions and take the sequence of the smallest value produced by each of these functions on the input as the MinHash sketch of that input. This sequence of values (which itself contains η members) reflects the smallest value the input set will produce when processed by each function. To compare two sets, we can generate their respective MinHash sketches and count the number of identical members, considering their positions in the order. The higher the similarity between the two sets, the greater the likelihood that their sketches will share more identical members. This increased likelihood stems from their similar behavior across the hash functions, which raises the probability of obtaining the same minimum member. It can be shown that the expected value of the equality between the MinHash sketches of two sets equals their Jaccard similarity.

### 1.2 MinHash and LSH in bioinformatics

The use of locality-sensitive hash (LSH) functions in bioinformatics has grown significantly in recent years, expanding to areas like motif finding, genome assembly, and alignment.

#### 1.2.1 Motif finding

MinHash was originally applied to motif finding, where a motif is a nucleotide sequence that identifies specific genomic regions. According to Buhler (2001), this involves finding all pairs of *k*-mers within a set *C* of sequences totaling length *N* that differ by no more than *r* letters. By setting r=0, the algorithm can identify identical shingles.

The algorithm generates a MinHash sketch for each *k*-length shingle in the genome, then groups identical sketches. Within these groups, it searches for shingles that differ by fewer than *r* letters. Although initial false negatives may impact speed, the algorithm remains efficient and practical overall.

Expanding on this method, Buhler and Tompa (2002) proposed identifying shingles with similar sketches as potential motifs and verifying them within the input sequences. Similarly, Wijaya *et al.* (2006) applied this technique to motif finding, utilizing a different verification algorithm.

#### 1.2.2 Genome assembly

Genome assembly is a fundamental task in bioinformatics, crucial for creating reference genomes that underpin various studies. Assembly errors can cause significant inaccuracies in later research Denton *et al.* (2014). One major challenge in genome assembly is the short length of sequencing reads. The introduction of MHAP Berlin *et al.* (2015), which uses MinHash with third-generation sequencing reads, addressed this by enhancing the accuracy of genome assemblies, despite the higher error rates associated with longer reads.

MHAP treats each read as a set of shingles, generates MinHash sketches, and uses these sketches in the assembly process. This method partitions MinHash sketches, finds the similar sketches in each part, and considers them as neighbors in the assembly process. Koren *et al.* (2017) extended MHAP to encompass all read types, incorporating a weighting system where shingle weights are inversely related to their frequency, a concept first introduced by Chum *et al.* (2008).

#### 1.2.3 Alignment

Genome alignment, which involves determining how and where a read aligns within the genome, becomes particularly challenging with third-generation sequencing. While these technologies generate long reads that offer more comprehensive coverage, their higher error rates pose significant difficulties. These errors complicate the task for traditional alignment tools, which rely on identifying seeds—short, identical segments between reads and the reference genome—that can be difficult to locate amidst the prevalent errors Chaisson and Tesler (2012); Li (2013).

Jain *et al.* (2017) utilized LSH for alignment, primarily focusing on identifying the genomic segment to which the reads belong, rather than achieving precise alignment. This study proposes an approximation algorithm that searches for long reads by generating MinHash sketches for both the reads and segments of the reference genome, subsequently matching these sketches to identify potential segments.

However, using MinHash for precise read alignment presents challenges due to its limitations in pinpointing the exact location within the genome. Additionally, the substantial size discrepancy between the genome and the read further impedes accuracy. To address this issue, Popic and Batzoglou (2017) introduced a sliding window approach to calculate MinHash sketches across the genome. This method generates fingerprints that are stored in hash tables, allowing the read’s fingerprint to be compared against these tables to identify potential matches.

### 1.3 Bisulfite sequencing

DNA methylation, a key epigenetic mechanism in vertebrates, involves the attachment of a methyl group to the carbon 5 of cytosine, forming 5-methylcytosine Weber *et al.* (2005). This process is crucial in imprinting, differentiation, development, inflammation, transcriptional silencing, and diseases such as cancer Varriale (2014). Analyzing methylation data therefore provides valuable insights into vertebrate cellular regulation.

Methylated cytosines primarily occur at CpG sites, where cytosine is adjacent to guanine. In contrast, CpA, CpT, and CpC sites are generally unmethylated Weber *et al.* (2005). CpG sites are often depleted in vertebrate genomes due to the deamination of methylated cytosines to uracil, leading to C-to-T conversions during DNA replication Hayatsu (2008). CpG islands, genomic regions with a higher density of CpGs and GC content, are typically hypomethylated, whereas isolated CpGs outside these islands tend to be hypermethylated Weber *et al.* (2005).

DNA methylation levels are measured using techniques such as methyl-DNA immunoprecipitation (MeDIP), methylation-sensitive restriction enzymes (MSREs), and bisulfite sequencing (BS) Kurdyukov and Bullock (2016). BS, when paired with next-generation sequencing (NGS), is preferred for its single-base resolution. BS treatment converts unmethylated cytosines to uracils, which are then converted to thymines during PCR, enabling the distinction between methylated (C) and unmethylated (T) cytosines by comparing sequenced reads to the reference genome.

Aligning NGS reads to the reference genome is a key challenge in analyzing BS data. Conventional short-read aligners like BWA Li and Durbin (2009) and Bowtie2 Langmead and Salzberg (2012) treat C-to-T and G-to-A conversions as mismatches, complicating alignment due to the frequent mismatches resulting from bisulfite treatment. Specific aligners for bisulfite sequencing reads have been developed, often by adapting the reads or the reference genome to allow conventional aligners to function without modification.

Two primary strategies address the C-to-T conversion issue. The first is three-letter alignment, where all Cs in both the reference and reads are converted to Ts, eliminating bisulfite-induced mismatches and enabling the use of conventional aligners Feng *et al.* (2011). Bismark Krueger and Andrews (2011) implements this by creating indices where Cs are converted to Ts and Gs to As, aligning reads to these indices. However, this method loses the distinction between Cs and Ts, causing multiple alignment hits and reducing uniquely aligned reads Bock (2012). An extended two-letter approach further increases information loss by converting both Cs to Ts and Gs to As Gong *et al.* (2022).

The second strategy, wildcard alignment, replaces all Cs in the reference with Y (representing C or T), allowing Cs and Ts in reads to align with reference Ys. BSMAP Xi and Li (2009) uses a hash table to map all k-mers and their C-to-T variations in the reference genome. While this avoids information loss, it biases alignment toward hypermethylated regions, as reads from these regions align more uniquely, leading to systematic overestimation of methylation ratios Bock (2012).

### 1.4 Our work

While the use of LSH functions has become commonplace in bioinformatics, their application to problems such as bisulfite sequencing—where the changes between the read and the reference genome result from systematic (C-to-T) but non-uniform alterations across the genome—has been less prevalent. This is because LSH, using a fixed metric, applies the same approach uniformly across the entire dataset.

In this paper, we introduce Multi-Metric locality-sensitive hashing (M2LSH), a generalization of LSH that employs multiple metrics for comparison, enhancing its effectiveness in more complex scenarios. Building on M2LSH, we present M3Hash, an extension that integrates multiple metrics into MinHash.

To further demonstrate the efficacy of using multiple metrics, we developed the BisHash tool for alignment in bisulfite sequencing. Unlike previous methods, such as the three-letter and wild-card approaches, BisHash does not confine itself to specific genomic regions. Our evaluation shows that although BisHash operates more slowly, it achieves higher accuracy in both standard and complex situations.

## 2 Methods

### 2.1 Formal definition of LSH and MinHash

Based on the definition provided in Indyk and Motwani (1998); Gionis *et al.* (1999), for a space *S* and a metric distance function *d*, a locality-sensitive hash function family $H=(h_{1},h_{2},\ldots ,h_{\eta })$ is introduced, where each function is defined as $h_{i}:S\to B$, and *B* is an arbitrary set. Additionally, two threshold values $\sigma_{1}$ and $\sigma_{2}$ exist such that $\sigma_{2}>\sigma_{1}$, along with two probability values $p_{1}$ and $p_{2}$, where $p_{1}>p_{2}$. These values are such that for any two inputs *a* and *b* in *S*, the following conditions are satisfied:

$$d(a,b)\leq \sigma_{1}\Rightarrow P_{H}(h_{i}(a)=h_{i}(b))\geq p_{1}$$

 

$$d(a,b)\geq \sigma_{2}\Rightarrow P_{H}(h_{i}(a)=h_{i}(b))\leq p_{2}$$

Assuming *h* is a hash function that maps elements of a universal set $M=(m_{1},m_{2},\ldots ,m_{\mu })$ to a metric space, and considering a set X⊂M, we define h(X)={h(x)|x∈X} and min(h(X)) as the minimum value within h(X).

In this context, if we have η hash functions represented as $\left(h_{1},h_{2},\ldots ,h_{\eta }\right)$ such that $h_{i}:M\to N$, then the family

$$H=(\min(h_{1}),\min(h_{2}),\ldots ,\min(h_{\eta }))$$

constitutes a locality-sensitive hash, known as MinHash. Consequently, if $A=(m_{a_{1}},m_{a_{2}},\ldots ,m_{a_{\alpha }})$ and $B=(m_{b_{1}},m_{b_{2}},\ldots ,m_{b_{\beta }})$ are two subsets of *M*, then $\min(h_{i}(A))=\min(h_{i}(B))$ only if the element with the minimum hash value for $h_{i}$ in A∪B is present in both *A* and *B*. Mathematically:

$$P_{H}(\min(h_{i}(A))=\min(h_{i}(B)))=\frac{\left|A\cap B\right|}{\left|A\cup B\right|}=J(A,B)=1-\text{JDIS}(A,B)$$

This expression indicates that the probability that the minimum hash values of two sets *A* and *B* are equal corresponds to their Jaccard similarity J(A,B), which is defined as the ratio of the intersection to the union of the two sets.

### 2.2 Multi-Metric locality sensitive hashing

Identifying the similarity between two proteins is a critical task in proteomics studies, as it provides insights into the downstream functions of proteins. In recent years, various approaches have been developed to determine the three-dimensional structure Jumper *et al.* (2021) or functionality of proteins. However, assessing protein similarity based on three-dimensional structure or overall function remains challenging, as two proteins can perform the same function despite having different three-dimensional structures. This challenge arises because different domains within a protein contribute distinct roles, and a specific function of a protein domain can often be achieved with various three-dimensional shapes, without requiring identical structures. Therefore, to evaluate the similarity between two proteins, it is essential to analyze their parts using multiple metrics.

Consider, for example, that the similarity of each part of two proteins can be assessed using various metrics, such as three-dimensional structure $\left(d_{s}\right)$, the total weight of amino acids $\left(d_{w}\right)$, or hydrophobicity level $\left(d_{h}\right)$. Let us represent two proteins, *a* and *b*, as follows:

$$\begin{array}{c} a=\{m_{P_{i_{1}}},\ldots ,m_{P_{i_{\alpha }}}\}\\ b=\{m_{P_{j_{1}}},\ldots ,m_{P_{j_{\beta }}}\} \end{array}$$

where:

$$m_{x}\in M=\{m_{1},\ldots ,m_{n_{M}}\}$$

and *M* denotes the reference set of all existing domains. Now, if we consider an arbitrary *z* (e.g., the number of possible domain types) and partition *M* into $Z_{1},\ldots ,Z_{z}$, we define *z* metrics $d_{di_{1}},\ldots ,d_{di_{z}}$, where each $d_{di_{x}}\in \{d_{s},d_{h},d_{w}\}$ corresponds to a specific metric with a threshold $\delta_{1}$ that defines the desired level of similarity. If the following conditions hold:

$$\begin{array}{c} d_{di_{1}}(a\cap Z_{1},b\cap Z_{1})\leq \delta_{1}\\ d_{di_{2}}(a\cap Z_{2},b\cap Z_{2})\leq \delta_{1}\\ \vdots \\ d_{di_{z}}(a\cap Z_{z},b\cap Z_{z})\leq \delta_{1} \end{array}$$

this indicates that for each domain type, there exists a metric that, when applied, reveals similarity between the corresponding parts of the two proteins associated with that domain type. This approach ensures that each domain type is compared with its appropriate metric, enabling a comprehensive, multi-metric assessment of protein similarity.

Definition: A Multi-Metric locality-sensitive hash function for a reference set *M*, given an arbitrary partition of it as $\left\{Z_{1},Z_{2},\ldots ,Z_{z}\right\}$ and the space *S* (the set of all subsets of *M*) and the metric family $D=\{d_{1},d_{2},\ldots ,d_{\Delta }\}$ where $d_{i}$’s are defined on *S*, is a family $H=\{h_{1},h_{2},\ldots ,h_{\eta }\}$ where $h_{i}:S\to B$ and *B* is an arbitrary set. Additionally, there exist two threshold values $\delta_{1}$ and $\delta_{2}$ such that $\delta_{1}>\delta_{2}$ and two probability values $p_{1}>p_{2}$ such that for any two inputs a,b∈S and an order $dz=(d_{i_{1}},d_{i_{2}},\ldots ,d_{i_{z}})$ such that $d_{i_{j}}\in D$, if we have:

$$\begin{array}{c} d_{i_{1}}(a\cap Z_{1},b\cap Z_{1})\leq \delta_{1},\\ d_{i_{2}}(a\cap Z_{2},b\cap Z_{2})\leq \delta_{1},\\ \vdots \\ d_{i_{z}}(a\cap Z_{z},b\cap Z_{z})\leq \delta_{1}, \end{array}$$

then:

$$P_{H}(h_{i}(a)=h_{i}(b))\geq p_{1}$$

and if for any *dz* one of the following conditions holds:

$$\begin{array}{c} d_{i_{1}}(a\cap Z_{1},b\cap Z_{1})\geq \delta_{2},\\ d_{i_{2}}(a\cap Z_{2},b\cap Z_{2})\geq \delta_{2},\\ \vdots \\ d_{i_{z}}(a\cap Z_{z},b\cap Z_{z})\geq \delta_{2}, \end{array}$$

then:

$$P_{H}(h_{i}(a)=h_{i}(b))\leq p_{2}.$$

### 2.3 Multi-metric Jaccard similarity

The Jaccard distance measures dissimilarity between two sets, calculated as one minus the Jaccard similarity. The Jaccard similarity is defined within a space containing a reference set. When considering different metrics for the reference elements, it necessitates a redefinition of the Jaccard similarity. Thus, the Multi-Metric Jaccard similarity on a reference set *M*, with an arbitrary partition $\left\{Z_{1},Z_{2},\ldots ,Z_{z}\right\}$ and a set of metrics $D=\{d_{1},d_{2},\ldots ,d_{\Delta }\}$, is defined as follows:

$$\begin{array}{c} \mathrm{JSI}M_{Z,D}(A,B)=\\ \frac{\underset{\left\{d_{\pi_{i}}|1\leq \pi_{i}\leq \Delta \wedge 1\leq i\leq z\right\}}{\max}\sum_{i=1}^{z}|(A\cap Z_{i})\cap_{d_{\pi_{i}}}(B\cap Z_{i})|}{\left|A\cup B\right|} \end{array}$$

To compute Multi-Metric Jaccard similarity, we assess two sets under each subset partition using the metric that yields the highest similarity, then sum these values.

### 2.4 Multi-metric MinHash

Consider a reference set $M=(m_{1},m_{2},\ldots ,m_{z*\mu })$, and define $\zeta =\{\zeta_{1},\zeta_{2},\ldots ,\zeta_{z}\}$ where:

$$\zeta_{i}=\{m_{(i-1)*\mu +1},m_{(i-1)*\mu +2},\ldots ,m_{(i-1)*\mu +\mu }\}$$

and Δ hash functions $\left(h_{1},h_{2},\ldots ,h_{\Delta }\right)$ where $h_{i}:M\to N$ and:

$$\forall m_{\upsilon },m_{\omega }\in M:d_{i}(\{m_{\upsilon }\})=d_{i}(\{m_{\omega }\})\Rightarrow h_{i}(\{m_{\upsilon }\})=h_{i}(\{m_{\omega }\})$$

then the family:

(1) $$H=(h_{1,1}^{\prime },h_{1,2}^{\prime },\ldots ,h_{1,\Delta }^{\prime },h_{2,1}^{\prime },h_{2,2}^{\prime },\ldots ,h_{2,\Delta }^{\prime },\ldots ,h_{z,1}^{\prime },h_{z,2}^{\prime },\ldots ,h_{z,\Delta }^{\prime })h_{i,j}^{\prime }(A)=\underset{x\in A\cap \zeta_{i}}{\min}(h_{j}(x))$$

is a multi-metric locality-sensitive hash function, termed multi-metric MinHash. We also define

$$\begin{array}{c} H(A)=(h_{1,1}^{\prime }(A),h_{1,2}^{\prime }(A),\ldots ,h_{1,\Delta }^{\prime }(A),\\ h_{2,1}^{\prime }(A),h_{2,2}^{\prime }(A),\ldots ,h_{2,\Delta }^{\prime }(A),\\ \ldots ,\\ h_{z,1}^{\prime }(A),h_{z,2}^{\prime }(A),\ldots ,h_{z,\Delta }^{\prime }(A)) \end{array}$$

as the sketch of *A*.

To prove that Multi-Metric MinHash is based on Multi-Metric Jaccard similarity, consider two sets $A=(m_{a_{1}},m_{a_{2}},\ldots ,m_{a_{\alpha }})$ and $B=(m_{b_{1}},m_{b_{2}},\ldots ,m_{b_{\beta }})$ in the space *M*. Define:

$$\begin{array}{c} \Xi_{i,j}(A,B)=\{\begin{array}{cc} 1, & h_{i,j}^{\prime }(A)=h_{i,j}^{\prime }(B)\vee A\cap \zeta_{i}=B\cap \zeta_{i}=\emptyset \\ 0, & \text{otherwise} \end{array}\\ \Xi_{i}(A,B)=\{\begin{array}{cc} 1, & \exists j\Xi_{i,j}(A,B)=1\\ 0, & \text{otherwise} \end{array}\\ \Xi^{s}(A,B)=\frac{1}{z}\sum_{i=1}^{z}\Xi_{i}(A,B) \end{array}$$

In this case, if we consider the partition ζ for the Multi-Metric Jaccard similarity as $\left\{\zeta_{1},\zeta_{2},\ldots ,\zeta_{z}\right\}$, we will have:

$$\begin{array}{c} E_{H}(\Xi_{i}(A,B))=\\ E_{H}(\exists j\Xi_{i,j}(A,B)=1)=\\ 1-\prod_{j=1}^{\Delta }(1-E_{H}(\Xi_{i,j}(A,B)))=\\ 1-\prod_{j=1}^{\Delta }(1-E(h_{i,j}^{\prime }(A)=h_{i,j}^{\prime }(B)))=\\ 1-\prod_{j=1}^{\Delta }(1-E(\underset{x\in A\cap \zeta_{i}}{\min}(h_{j}(x))=\underset{x\in B\cap \zeta_{i}}{\min}(h_{j}(x))))=\\ 1-\prod_{j=1}^{\Delta }\left(1-\frac{\left|(A\cap \zeta_{i})\cap_{d_{j}}(B\cap \zeta_{i})\right|}{\left|(A\cap \zeta_{i})\cup (B\cap \zeta_{i})\right|}\right)=\\ E(\underset{d_{j}}{\max}\frac{\left|(A\cap \zeta_{i})\cap_{d_{j}}(B\cap \zeta_{i})\right|}{\left|(A\cap \zeta_{i})\cup (B\cap \zeta_{i})\right|})=\\ \frac{E(\underset{d_{j}}{\max}|(A\cap \zeta_{i})\cap_{d_{j}}(B\cap \zeta_{i})|)}{E(|(A\cup B)\cap \zeta_{i}|)}=\\ \frac{E(\underset{d_{j}}{\max}|(A\cap \zeta_{i})\cap_{d_{j}}(B\cap \zeta_{i})|)}{\frac{E(|A\cup B|)}{z}}=\\ z\cdot E\left(\underset{d_{j}}{\max}\frac{\left|(A\cap \zeta_{i})\cap_{d_{j}}(B\cap \zeta_{i})\right|}{\left|A\cup B\right|}\right) \end{array}$$

Therefore:

$$\begin{array}{c} E_{H}\left(\Xi^{s}(A,B)\right)=\\ \frac{1}{z}\sum_{i=1}^{z}E_{H}\left(\Xi_{i}(A,B)\right)=\\ \frac{1}{z}\sum_{i=1}^{z}z\cdot E\left(\underset{d_{j}}{\max}\frac{\left|(A\cap \zeta_{i})\cap_{d_{j}}(B\cap \zeta_{i})\right|}{\left|A\cup B\right|}\right)=\\ E\left(\sum_{i=1}^{z}\underset{d_{j}}{\max}\frac{\left|(A\cap \zeta_{i})\cap_{d_{j}}(B\cap \zeta_{i})\right|}{\left|A\cup B\right|}\right)=\\ E\left(\frac{\sum_{i=1}^{z}\underset{d_{j}}{\max}|(A\cap \zeta_{i})\cap_{d_{j}}(B\cap \zeta_{i})|}{\left|A\cup B\right|}\right)=\\ E\left(\frac{\underset{\left\{d_{\pi_{i}}|\leq \pi_{i}\leq \Delta \wedge 1\leq i\leq z\right\}}{\max}\sum_{i=1}^{z}|(A\cap \zeta_{i})\cap_{d_{\pi_{i}}}(B\cap \zeta_{i})|}{\left|A\cup B\right|}\right)=\\ E\left(\mathrm{JSI}M_{\zeta ,D}(A,B)\right) \end{array}$$

Operationally, to compute the Multi-Metric MinHash, it suffices to evaluate the value of *H* for a set. To determine the closeness of two sets, we check whether for each *i*, there exists at least one *j* such that $h_{i,j}^{\prime }(A)=h_{i,j}^{\prime }(B)$. It is worth noting that in some cases, due to the small size of the input set, the value $h_{i,j}^{\prime }(A)$ may not be defined. In such instances, equality occurs when this value is also undefined for set *B*. If we can partition the reference set in a manner that reflects a real partition, the results are better, however partitioning it randomly also works.

### 2.5 BisHash: multi-metric locality sensitive hashing for bisulfite alignment

C-to-T changes in bisulfite sequencing reads complicate their alignment with the reference genome. Traditional methods that verify nucleotide sequence equality are inadequate for detecting similarities in bisulfite sequencing alignment. Similarly, relying solely on a metric that considers C and T as equal is insufficient. Since some similarities between the read and reference genome are detected by the first metric and others by the second, using both metrics simultaneously is crucial for accurate alignment. Multi-Metric MinHash provides a solution to this challenge.

#### 2.5.1 Segment selection

To apply Multi-Metric MinHash, we first divide the reference genome into segments of length *T*. To ensure that each read is fully contained within at least one segment, we select consecutive segments with an overlap of at least τ, where τ is equal to the maximum read length. The segment length *T* should be chosen to be comparable to the read length to optimize coverage and accuracy. This setup enables the identification of the segments most similar to each read based on both metrics, facilitating the precise determination of the read’s location within the most similar segments.

Formally, let $d_{1}$ represent the distance between two sequences without alterations, and $d_{2}$ represent the distance considering the equality of cytosine and thymine. The reference set *M* consists of all possible k-mers, where the k-mers present in each read or segment represent its set. To define ζ, we partition the reference set based on the first κ letters of each k-mer. Since one of the first κ letters in the intersection between the set related to the read and the members of ζ might be a cytosine converted to thymine, we use a 3-letter method for this partition, treating cytosine and thymine as a single letter. Consequently, we have $z=3^{\kappa }$.

Using this approach, we compute the minimum Multi-Metric hash for each read and segment to identify the segment with the highest similarity to the read. To achieve this, we construct a tree-based data structure (utilizing a C++ “map” structure) to efficiently track which segments contain specific values in their sketches. For a given read, we then iterate over its sketch values and check which segments contain each of these values. A “map” structure is used to count the occurrences of matches for each segment. The segment with the highest match count is considered the most similar to the read. This method ensures efficient similarity detection while minimizing unnecessary computations.

**Figure 1.**
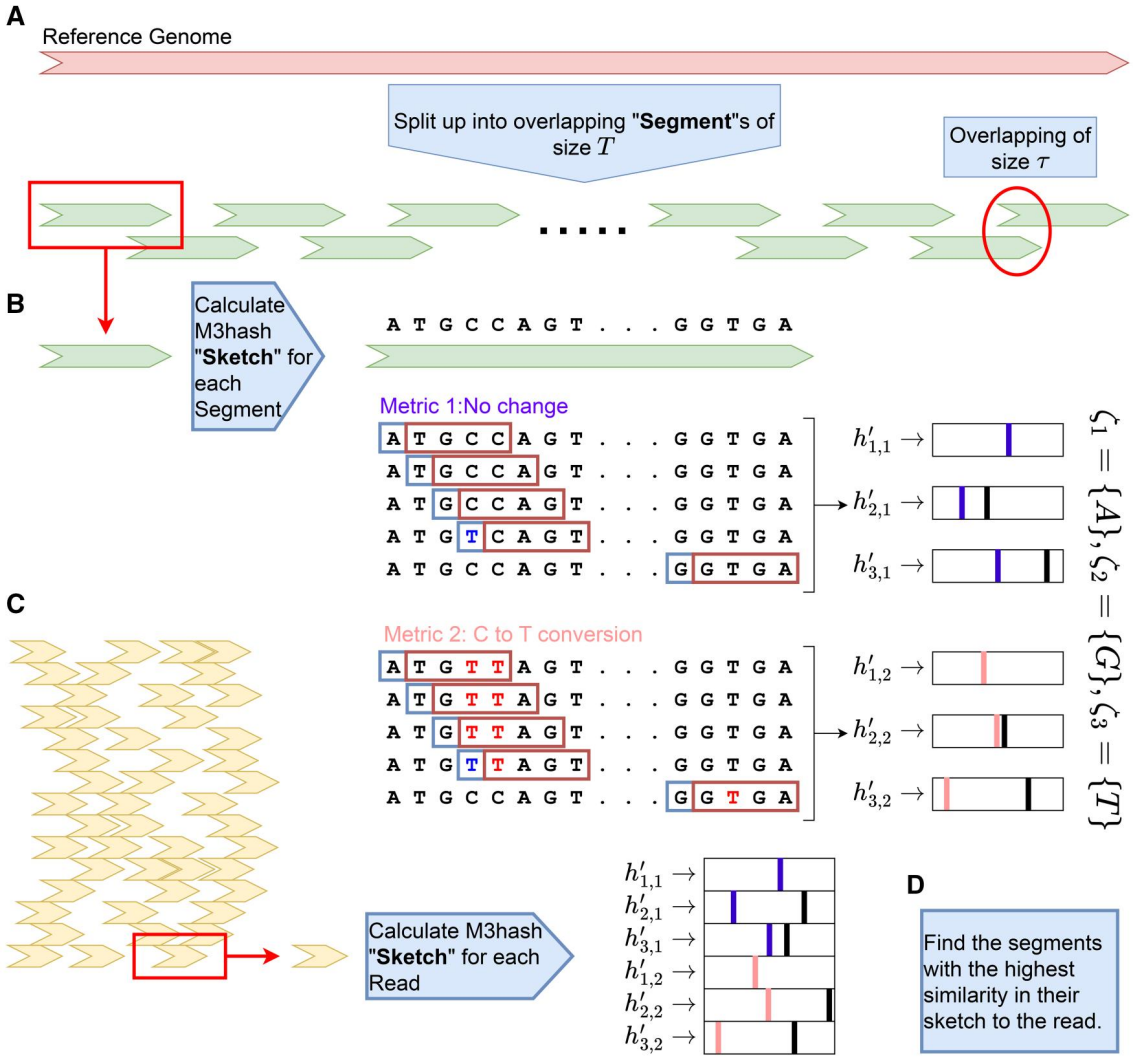
Segmentation selection. (A) The reference genome is divided into overlapping segments of the same length *T*. Each segment overlaps with the next segment by τ, which is greater than the read length. (B) We generate M3Hash sketches for all segments using two metrics. (C) We generate the M3Hash for each read in the same manner as for each segment. (D) We compare the read sketch with all segment sketches to find the most similar segments to the read. We repeat steps (B) and (C) for Guanine-to-Adenine conversion and find the most similar segments to the read in this context as well.

If multiple segments meet the defined similarity threshold, they are all considered similar and are further analyzed in the next step. We repeat these steps for Guanine-to-Adenine conversion and identify the most similar segments to the read in this context as well (Fig. 1).

#### 2.5.2 Context-aware read to segment extension

As previously elucidated, BisHash partitions each reference genome into equitably sized windows, termed “segments,” to discern the optimal genomic segment for read mapping. The evaluation of segments is accomplished using Multi-Metric MinHash, resulting in a compilation of the most highly ranked segments. Subsequently, a concerted effort ensues to establish a congruence between the segment and the reader. To facilitate this alignment process, a dynamic programming algorithm has been meticulously devised to determine the optimal match between the read and the segment. While this algorithm follows the foundational principles of Smith-Waterman, it differs through several notable features.

The scoring paradigm employed is tailored to the nuances of bisulfite sequencing. In cases where the read aligns with the segment using C-to-T indexes, a zero penalty is imposed for genomic cytosines corresponding to read thymines. However, genomic thymines aligned with read cytosines are classified as mismatches. This distinctive approach differentiates BisHash from aligners that use two- or three-letter schemes or wildcard methodologies.

For reads aligning with the segment using G-to-A indexes, a similar penalty framework is applied. Genomic guanines aligning with read adenines incur penalties in a manner akin to the aforementioned scheme.

## 3 Results

The proposed BisHash tool, developed with the Multi-Metric MinHash (M3Hash) technique, was rigorously evaluated against established bisulfite sequencing alignment tools: ARYANA-BS, BWA-meth, Bismark, BSBolt Farrell *et al.* (2021), and abismal de Sena Brandine and Smith (2021). The tool BSMAP was excluded from the detailed comparison as it is unable to align reads longer than 144 bp, which limits its utility for the simulated dataset of 300 bp reads. The assessment was conducted under six distinct scenarios (Table 1):

**Table 1.** Summary of test scenarios.

Test ID	Methylation Type	# Reads	Threading
Test 1	Normal	1 000 000	Single-threaded
Test 2	Random	1 000 000	Single-threaded
Test 3	Normal	200 000	Single-threaded
Test 4	Random	200 000	Single-threaded
Test 5	Normal	1 000 000	8 threads
Test 6	Random	1 000 000	8 threads

- Test 1 and Test 2: Standard tests using 1 million reads, under normal and random methylation settings, respectively.
- Test 3 and Test 4: Same conditions as Test 1 and Test 2, but using only 200 000 reads to investigate scalability.
- Test 5 and Test 6: Identical to Test 1 and Test 2, respectively, but executed with 8 threads to evaluate multithreading performance.

In Tests 2, 4, and 6, methylation was simulated in a random pattern across the genome to reflect real-world biological complexities, such as those seen in cancer, where methylation patterns are often unpredictable. Assessing aligner performance under these conditions provides insight into their robustness when dealing with highly variable landscapes.

All tools were tested under consistent conditions to ensure a fair comparison. The simulations were performed using hg38 as the reference genome, with bisulfite-treated reads generated by the ARYANA simulator. Each tool was run with its default parameters on an AMD Ryzen 5 3600X CPU with a maximum memory allowance of 48 GB. Tests 1 to 4 were executed in single-threaded mode, while Tests 5 and 6 used 8 threads. The reads used in the simulation were 300 bp in length. A total of 1 million reads were used in Tests 1, 2, 5, and 6, while 200 000 reads were used in Tests 3 and 4. For a read to be considered correctly aligned, it had to be within 1 bp distance from the simulated position. The versions of the tools used were as follows: ARYANA-BS (aryana_bs branch) Nikaein *et al.* (2024), BWA-meth (v0.2.2, via Bioconda) Pedersen *et al.* (2014), Bismark (v0.23.0, via Bioconda) Krueger and Andrews (2011), BSBolt (v1.4.8 via Bioconda), abismal (v3.1.1 master branch), and BisHash. The script and parameters used for these simulations are available in the “simulation” folder of the GitHub repository linked to this project.

**Figure 2.**
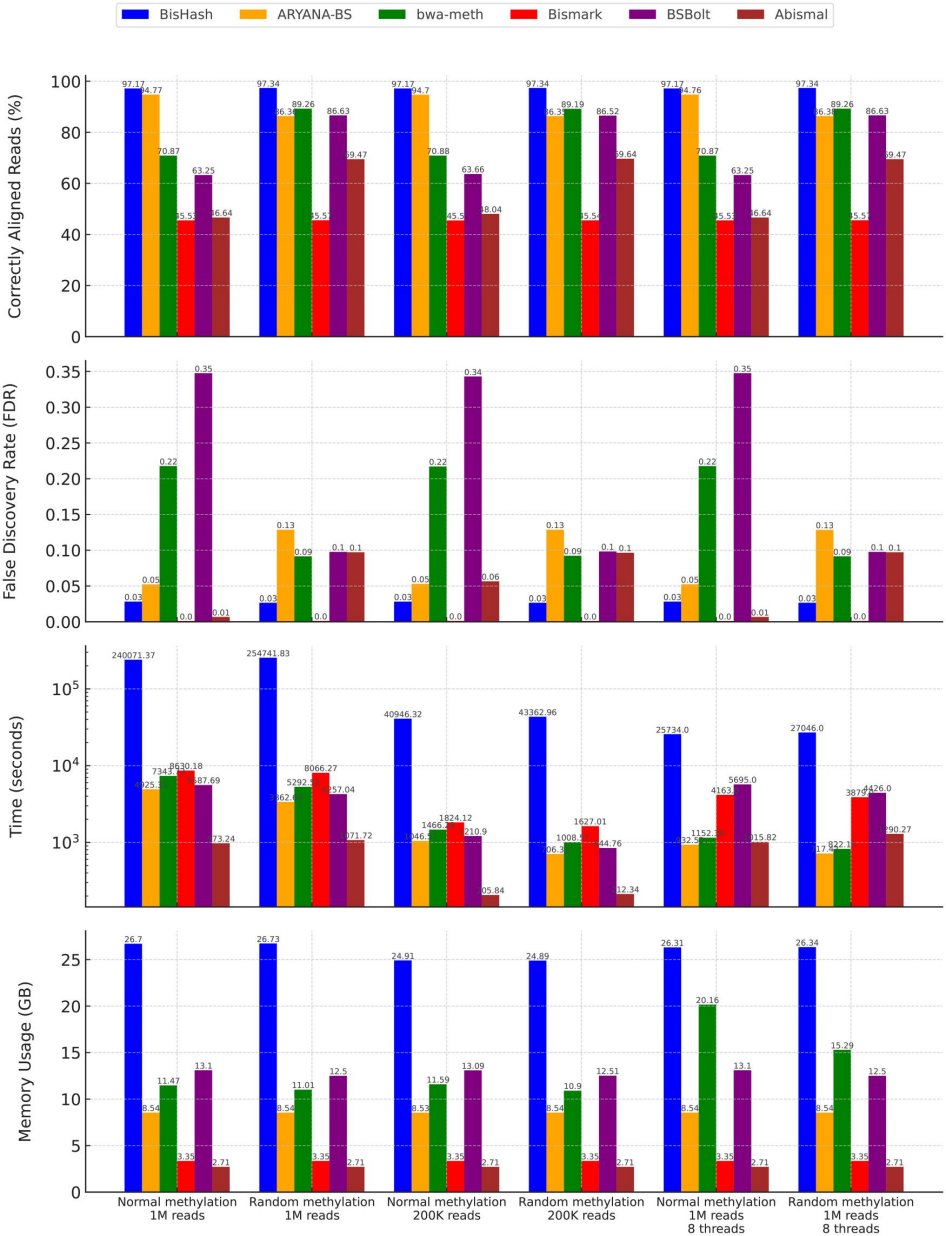
Comparative performance of bisulfite sequencing alignment tools across six test scenarios: (A) normal methylation with 1 million reads (single-threaded), (B) random methylation with 1 million reads (single-threaded), (C) normal methylation with 200,000 reads (single-threaded), (D) random methylation with 200,000 reads (single-threaded), (E) normal methylation with 1 million reads (8 threads), and (F) random methylation with 1 million reads (8 threads). Metrics evaluated include correctly aligned reads, false discovery rate (FDR), Time Efficiency (logarithmic scale), and memory usage.

Figure 2 provides a visual summary and comparative analysis of the tools across four key aspects in all test scenarios.

### 3.1 Correctly aligned reads

Each tool’s ability to correctly align reads was assessed by comparing alignment positions to the ground truth. BisHash demonstrated superior accuracy in all test scenarios, achieving the highest percentage of correctly aligned reads. Notably, in the normal simulation scenarios (Tests 1, 3, and 5), BisHash outperformed all other tools, including ARYANA-BS and BWA-meth, by a significant margin. This shows that BisHash’s Multi-Metric approach effectively enhances alignment precision, particularly in challenging genomic regions impacted by bisulfite-induced modifications.

### 3.2 False discovery rate

False discovery rate (FDR) was calculated to evaluate the proportion of incorrect alignments. The FDR is defined by the formula:

$$\text{FDR}=\frac{\text{Count}\;\text{of}\;\text{incorrectly}\;\text{mapped}\;\text{reads}}{\text{Count}\;\text{of}\;\text{total}\;\text{mapped}\;\text{reads}}$$

BisHash consistently exhibited the lowest FDR across all test scenarios, reaffirming its accuracy and reliability. Traditional tools, particularly BWA-meth, showed higher FDRs, indicating a greater number of incorrect alignments. This suggests that the single-metric approaches used by these tools may not adequately account for the complexities introduced by bisulfite treatment, leading to a higher likelihood of false positives.

### 3.3 Time efficiency

The time each tool needed to process reads was measured. BisHash required a longer runtime compared to ARYANA-BS and abismal, which is expected given its more sophisticated Multi-Metric algorithm. However, the additional time cost is justified by substantial gains in accuracy and reduced FDR. Tests 3 and 4 showed that reducing the number of reads to 200 000 results in proportionally reduced runtimes, indicating that the time complexity of BisHash scales linearly with input size. Furthermore, the results of Tests 5 and 6, which were conducted using 8 threads, show a clear improvement in runtime for BisHash. Although it still does not fully match the speed of the fastest tools, the multithreaded implementation significantly narrows the performance gap. This indicates that BisHash can benefit meaningfully from parallelization and, with further optimizations, could reach practical runtime competitiveness in multicore environments. The time results are shown on a logarithmic scale to highlight the tradeoff between computational time and alignment accuracy.

### 3.4 Memory usage

Memory consumption is an important factor in large-scale genomic analyses. BisHash showed higher memory usage than other tools like Bismark but remained within acceptable limits given the test hardware configuration. The memory footprint of BisHash is a result of its advanced algorithmic complexity, which integrates multiple metrics to enhance alignment precision. Despite this, the memory usage was efficiently managed and did not pose significant constraints during the experiments.

### 3.5 Overall performance

Overall, BisHash, powered by the M3Hash algorithm, demonstrated significant improvements in read alignment accuracy and FDR reduction, albeit with increased time and memory requirements. These results suggest that BisHash is a highly effective tool for bisulfite sequencing analysis, offering a robust solution to the challenges posed by bisulfite-induced modifications in DNA sequences. Its superior accuracy makes the tool particularly valuable for high-precision applications, such as epigenetic studies and cancer genomics. Additionally, the results of Tests 5 and 6 indicate that BisHash scales efficiently with multithreading. Although its runtime does not yet match the fastest tools, the clear trend toward performance improvement with increased thread count highlights its potential for future optimization.

## 4 Discussion

Multi-Metric Locality-Sensitive Hashing (M2LSH) represents a significant advancement in bioinformatics by effectively addressing the limitations inherent to single-metric similarity estimation. By incorporating multiple metrics, M2LSH significantly enhances alignment accuracy, particularly beneficial in complex genomic datasets where regions evolve under diverse evolutionary pressures.

The Multi-Metric MinHash (M3Hash), developed based on M2LSH, exemplifies these improvements through BisHash, which consistently outperformed established bisulfite sequencing tools across all tested scenarios. A detailed breakdown analysis revealed BisHash’s superiority, particularly under challenging conditions involving random methylation patterns, mimicking biologically complex scenarios like cancer. In these tests, BisHash achieved the highest proportion of correctly aligned reads and maintained the lowest false discovery rates (FDR). This demonstrates its robustness in accurately handling datasets with unpredictable methylation landscapes.

Although BisHash showed increased computational time and higher memory consumption relative to some traditional methods, the performance gains justify these resource costs. Importantly, scalability tests demonstrated linear runtime scaling and significant runtime enhancements with multithreading, indicating promising avenues for optimization.

## References

[vbaf144-B1] Berlin K , Koren S, Chin C-S et al Assembling large genomes with single-molecule sequencing and locality-sensitive hashing. Nat Biotechnol 2015;33:623–30. 26006009 10.1038/nbt.3238

[vbaf144-B2] Bock C. Analysing and interpreting DNA methylation data. Nat Rev Genet 2012;13:705–19. 22986265 10.1038/nrg3273

[vbaf144-B3] Broder AZ. Identifying and filtering near-duplicate documents. In: *Annual Symposium on Combinatorial Pattern Matching*. Berlin: Springer, 2000, 1–10.

[vbaf144-B4] Buhler J. Efficient large-scale sequence comparison by locality-sensitive hashing. Bioinformatics 2001;17:419–28. 11331236 10.1093/bioinformatics/17.5.419

[vbaf144-B5] Buhler J , Tompa M. Finding motifs using random projections. J Comput Biol 2002;9:225–42. 12015879 10.1089/10665270252935430

[vbaf144-B6] Chaisson MJ , Tesler G. Mapping single molecule sequencing reads using basic local alignment with successive refinement (BLASR): application and theory. BMC Bioinform 2012;13:238. 10.1186/1471-2105-13-238 PMC3572422 22988817

[vbaf144-B7] Chum O , Philbin J, Zisserman A et al Near duplicate image detection: min-hash and tf-idf weighting. In: *Proceedings of the British Machine Vision Conference (BMVC)*, *Leeds, UK*, 2008, 812–15.

[vbaf144-B8] Denton JF , Lugo-Martinez J, Tucker AE et al Extensive error in the number of genes inferred from draft genome assemblies. PLoS Comput Biol 2014;10:e1003998. 25474019 10.1371/journal.pcbi.1003998 PMC4256071

[vbaf144-B9] de Sena Brandine G , Smith AD. Fast and memory-efficient mapping of short bisulfite sequencing reads using a two-letter alphabet. NAR Genom Bioinform 2021;3:lqab115. 34988438 10.1093/nargab/lqab115 PMC8693577

[vbaf144-B10] Farrell C , Thompson M, Tosevska A et al Bisulfite bolt: a bisulfite sequencing analysis platform. Gigascience 2021;10:giab033. 33966074 10.1093/gigascience/giab033 PMC8106542

[vbaf144-B11] Feng S , Rubbi L, Jacobsen SE et al Determining DNA methylation profiles using sequencing. In: Gustafson C, Heldin C-H (eds), High-Throughput Next Generation Sequencing. Berlin: Springer, 2011, 223–38. 10.1007/978-1-61779-089-8_16 21431774

[vbaf144-B12] Gionis A , Indyk P, Motwani R et al Similarity search in high dimensions via hashing. In: *Proceedings of the 25th International Conference on Very Large Data Bases (VLDB), Edinburgh, UK*, 1999, 518–29.

[vbaf144-B13] Gong W , Pan X, Xu D et al Benchmarking DNA methylation analysis of 14 alignment algorithms for whole genome bisulfite sequencing in mammals. Comput Struct Biotechnol J 2022;20:4704–16. 36147684 10.1016/j.csbj.2022.08.051 PMC9465269

[vbaf144-B14] Hayatsu H. Discovery of bisulfite-mediated cytosine conversion to uracil, the key reaction for DNA methylation analysis—a personal account. Proc Jpn Acad, Ser B 2008;84:321–30. 18941305 10.2183/pjab/84.321 PMC3722019

[vbaf144-B15] Indyk P , Motwani R. Approximate nearest neighbors: towards removing the curse of dimensionality. In: *Proceedings of the 30th Annual ACM Symposium on Theory of Computing, Dallas, TX*, 1998, 604–13. New York: ACM.

[vbaf144-B16] Jain C , Dilthey A, Koren S et al A fast approximate algorithm for mapping long reads to large reference databases. In: Sharan R (ed.), *Research in Computational Molecular Biology*. Berlin: Springer, 2017, 66–81. 10.1089/cmb.2018.0036 PMC6067103 29708767

[vbaf144-B17] Jumper J , Evans R, Pritzel A et al Highly accurate protein structure prediction with alphafold. nature 2021;596:583–9. 34265844 10.1038/s41586-021-03819-2 PMC8371605

[vbaf144-B18] Knuth DE. The Art of Computer Programming. Vol. 3: Sorting and Searching. 2nd edn. Boston: Addison-Wesley, 1998.

[vbaf144-B19] Koren S , Walenz BP, Berlin K et al Canu: scalable and accurate long-read assembly via adaptive k-mer weighting and repeat separation. Genome Res 2017;27:722–36. 28298431 10.1101/gr.215087.116 PMC5411767

[vbaf144-B20] Krueger F , Andrews SR. Bismark: a flexible aligner and methylation caller for bisulfite-seq applications. Bioinformatics 2011;27:1571–2. 21493656 10.1093/bioinformatics/btr167 PMC3102221

[vbaf144-B21] Kurdyukov S , Bullock M. DNA methylation analysis: choosing the right method. Biology (Basel) 2016;5:3. 26751487 10.3390/biology5010003 PMC4810160

[vbaf144-B22] Langmead B , Salzberg SL. Fast gapped-read alignment with bowtie 2. Nat Methods 2012;9:357–9. 22388286 10.1038/nmeth.1923 PMC3322381

[vbaf144-B23] Li H. Aligning sequence reads, clone sequences and assembly contigs with BWA-MEM. *arXiv*, https://arxiv.org/abs/1303.3997, 2013.

[vbaf144-B24] Li H , Durbin R. Fast and accurate short read alignment with burrows–wheeler transform. Bioinformatics 2009;25:1754–60. 19451168 10.1093/bioinformatics/btp324 PMC2705234

[vbaf144-B25] Nikaein H , Sharifi-Zarchi A, Afzal A et al Aryana-BS: context-aware alignment of bisulfite-sequencing reads. bioRxiv, 10.1101/2024.01.20.576080v1, 20 January 2024. PMC12281798 40691759

[vbaf144-B26] Pedersen BS , Eyring K, De S et al Fast and accurate alignment of long bisulfite-seq reads. arXiv, https://arxiv.org/abs/1401.1129, 2014, preprint: not peer reviewed.

[vbaf144-B27] Popic V , Batzoglou S. A hybrid cloud read aligner based on minhash and kmer voting that preserves privacy. Nat Commun 2017;8:15311. 28508884 10.1038/ncomms15311 PMC5440850

[vbaf144-B28] Varriale A. DNA methylation, epigenetics, and evolution in vertebrates: facts and challenges. Int J Evol Biol 2014;2014:475981. 24551476 10.1155/2014/475981 PMC3914449

[vbaf144-B29] Weber M , Davies JJ, Wittig D et al Chromosome-wide and promoter-specific analyses identify sites of differential DNA methylation in normal and transformed human cells. Nat Genet 2005;37:853–62. 16007088 10.1038/ng1598

[vbaf144-B30] Wijaya E , Rajaraman K, Brahmachary M et al A hybrid algorithm for motif discovery from DNA sequences. In: *Proceedings of the 3rd Asia-Pacific Bioinformatics Conference.* APBC, 2006.

[vbaf144-B32] Xi Y , Li W. BSMAP: whole genome bisulfite sequence mapping program. BMC Bioinformatics 2009;10:232–9. 19635165 10.1186/1471-2105-10-232 PMC2724425

